# A QTL for root growth angle on rice chromosome 7 is involved in the genetic pathway of *DEEPER ROOTING 1*

**DOI:** 10.1186/s12284-015-0044-7

**Published:** 2015-02-05

**Authors:** Yusaku Uga, Yuka Kitomi, Eiji Yamamoto, Noriko Kanno, Sawako Kawai, Tatsumi Mizubayashi, Shuichi Fukuoka

**Affiliations:** National Institute of Agrobiological Sciences, 2-1-2 Kannondai, Tsukuba, Ibaraki 305-8602 Japan; (Present address) NARO Institute of Vegetable and Tea Science, 360 Kusawa, Ano, Tsu, Mie 514-2392 Japan

**Keywords:** Chromosome segment substitution lines, *DRO1*, Drought avoidance, Quantitative trait locus, Root growth angle, Root system architecture

## Abstract

**Background:**

Root growth angle (RGA) is an important trait that influences the ability of rice to avoid drought stress. *DEEPER ROOTING 1* (*DRO1*), which is a major quantitative trait locus (QTL) for RGA, is responsible for the difference in RGA between the shallow-rooting cultivar IR64 and the deep-rooting cultivar Kinandang Patong. However, the RGA differences between these cultivars cannot be fully explained by *DRO1*. The objective of this study was to identify new QTLs for RGA explaining the difference in RGA between these cultivars.

**Results:**

By crossing IR64 (which has a non-functional allele of *DRO1*) with Kinandang Patong (which has a functional allele of *DRO1*), we developed 26 chromosome segment substitution lines (CSSLs) that carried a particular chromosome segment from Kinandang Patong in the IR64 genetic background. Using these CSSLs, we found only one chromosomal region that was related to RGA: on chromosome 9, which includes *DRO1*. Using an F_2_ population derived from a cross between Kinandang Patong and the Dro1-NIL (near isogenic line), which had a functional *DRO1* allele in the IR64 genetic background, we identified a new QTL for RGA (*DRO3*) on the long arm of chromosome 7.

**Conclusions:**

*DRO3* may only affect RGA in plants with a functional *DRO1* allele, suggesting that *DRO3* is involved in the *DRO1* genetic pathway.

**Electronic supplementary material:**

The online version of this article (doi:10.1186/s12284-015-0044-7) contains supplementary material, which is available to authorized users.

## Background

Water shortages, including some that may have been caused by recent global climate change, have occurred more frequently in recent years in agricultural land, and have decreased crop production (Pennisi 2008). Under drought conditions, water remains disproportionately in the deep soil layers. The survival of most terrestrial plants under drought depends on the ability of their roots to obtain water, and deeper rooting appears to be an efficient strategy for avoiding drought stress because it enables a plant to absorb more water from these deep soil layers (Yoshida and Hasegawa 1982; Fukai and Cooper 1995; Rich and Watt 2013). Thus, deeper rooting is thought to be a key trait in the improvement of drought avoidance in crops (Fukai and Cooper 1995; Manschadi et al. 2006; Kirkegaard et al. 2007). To enhance a crop’s ability to avoid drought stress, therefore, the introduction of genes responsible for deep rooting into shallow-rooting cultivars is a promising breeding strategy. Deep rooting in cereal crops is determined by the combination of a large root growth angle (RGA; the angle between the soil surface and the shallowest primary root) and long seminal and nodal roots (Abe and Morita 1994; Araki et al. 2002). In particular, RGA determines whether a plant develops shallow or deep rooting, because RGA predetermines the dominant direction of root elongation.

To use one or more quantitative trait loci (QTLs) for RGA to improve drought avoidance, QTL analyses have been performed in some crop species such as sorghum (Mace et al. 2012) and wheat (Hamada et al. 2012; Christopher et al. 2013). In rice (*Oryza sativa* L.), two QTLs for the root gravitropic response, which is an important component of RGA, have been detected on chromosomes 6 and 10 (Norton and Price 2009). Our research group has also reported three major rice QTLs for RGA, namely *DRO1* (*DEEPER ROOTING 1*), *DRO2*, and *qSOR1* (*quantitative trait locus for SOIL SURFACE ROOTING 1*), in three different mapping populations (Uga et al. 2011, 2012, 2013b). *DRO1* has been detected on chromosome 9 in recombinant inbred lines (IK-RILs) derived from a cross between the shallow-rooting cultivar IR64 and the deep-rooting cultivar Kinandang Patong (Uga et al. 2011). *DRO2* has been found on chromosome 4 in three F_2_ populations derived from crosses between each of three shallow-rooting cultivars (ARC5955, Pinulupot1, and Tupa729) and Kinandang Patong (Uga et al. 2013b). *qSOR1* has been fine-mapped to a position on chromosome 7 in advanced mapping lines derived from a cross between the lowland cultivar Gemdjah Beton, with soil-surface roots and Sasanishiki that lacks soil-surface roots (Uga et al. 2012). Further isolation and characterization of these RGA QTLs will be necessary to elucidate the genetic mechanisms associated with RGA and apply this knowledge to molecular breeding to control RGA.

Recently, our research group cloned and characterized *DRO1*, which functions downstream of the auxin signaling pathway and controls the gravitropic curvature of rice roots (Uga et al. 2013a). Under upland conditions with drought stress, a near-isogenic line (NIL) with a functional allele of *DRO1* introduced from Kinandang Patong (Dro1-NIL) had a significantly larger RGA and higher grain yield than the parental variety, IR64, which had a non-functional allele of *DRO1*. This demonstrates that a larger RGA can stabilize rice production under drought stress. Although we identified only *DRO1* in the IK-RILs in our previous study (Uga et al. 2011), *DRO1* alone cannot completely explain the differences in RGA between IR64 and Kinandang Patong. This suggests that one or more undetected QTLs for RGA existed in the IK-RILs. If we can discover one or more new QTLs for RGA, it seems likely that IR64’s ability to avoid drought could be improved by incorporating these QTLs, along with *DRO1*.

In mapping populations such as RILs, segregation of major QTLs like *DRO1* often masks the presence of minor QTLs. On the other hand, chromosome segment substitution lines (CSSLs) are efficient genetic materials for detection of minor QTLs because they can separate minor QTLs from major QTLs located in other chromosome regions (Fukuoka et al. 2010). In the present study, therefore, we developed new CSSLs and looked for previously undetected QTLs for RGA in them.

## Results

### Evaluation of deep rooting in the IK-CSSLs

**Figure 1:**
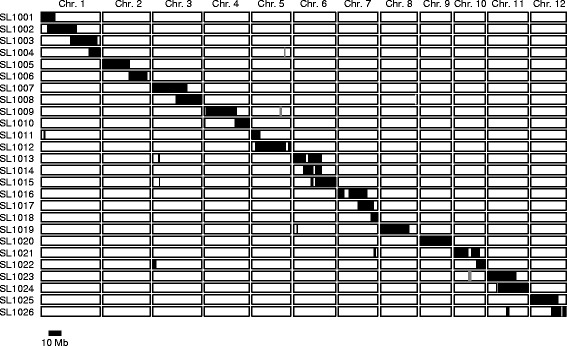
**Graphical genotypes of the 26 IK-CSSLs derived from a cross between IR64 and Kinandang Patong (see Additional file** 4 **for details).** White, black, and gray boxes indicate genotypes homozygous for IR64 alleles, those homozygous for Kinandang Patong alleles, and those heterozygous for these alleles, respectively.

**Figure 2:**
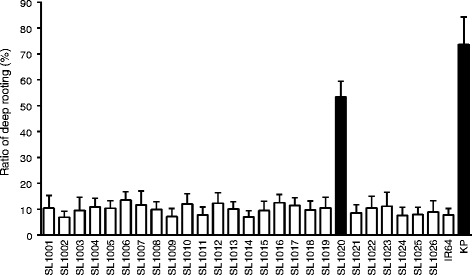
**Ratio of deep rooting for the 26 IK-CSSLs, IR64, and Kinandang Patong (KP).** Values are means + SD (*n* = 10). Black bars differ significantly between the IK-CSSL and IR64 (*p* < 0.001, Dunnett’s test).

We developed a set of 26 CSSLs derived from a cross between IR64 and Kinandang Patong (hereafter, IK-CSSLs) (Figure 1). Based on physical map positions of the 519 DNA markers used in this study, each chromosome was covered by one to four lines that carried overlapping segments, except for small regions on chromosomes 2, 5, 7, 8, and 10 that were not covered. A small segment on the long arm of chromosome 12 in line SL1026 remained heterozygous, because seeds of plants homozygous for Kinandang Patong at this region in the IR64 genetic background could not be obtained. Phenotypic evaluation of several physiological and aboveground morphological traits (heading date, culm length, and the length and number of panicles) in paddy fields revealed significant differences between several lines and IR64, suggesting that many QTLs segregated in this parental combination (Additional file 1: Figure S1). On the other hand, only plants of SL1020, which was homozygous for Kinandang Patong throughout chromosome 9 and which contained functional allele of *DRO1*, had a significantly larger mean ratio of deep rooting (RDR; 53.4%) than that of IR64 (7.8%), although the RDR in this line was significantly smaller than that in Kinandang Patong (73.7%) (Figure 2). RDR of the other lines, which included non-functional alleles of *DRO1*, did not differ significantly from that of IR64, and ranged from 6.9 to 13.5%.

### Detection of QTLs for deep rooting and RGA in the KD-F2 plants

**Figure 3:**
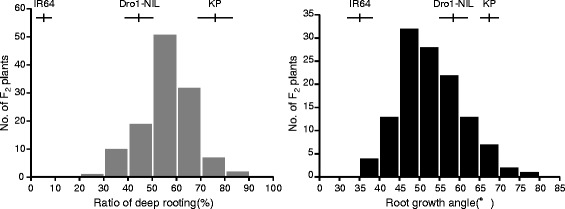
**Frequency distributions for the ratio of deep rooting and the root growth angle in the KD-F2 plants.** Vertical and horizontal lines above the bars indicate the mean and SD in the parental lines.

We developed an F_2_ population (KD-F2) derived from a cross between Kinandang Patong and Dro1-NIL because we did not find RGA QTLs in the IK-CSSLs having non-functional alleles of *DRO1*. When grown in the baskets, Dro1-NIL (RDR = 44.0%; RGA = 58.3°) had deeper roots than IR64 (RDR = 4.4%; RGA = 35.1°) but shallower roots than Kinandang Patong (RDR = 76.1%; RGA = 67.1°) (Figure 3). The RDRs in the KD-F2 plants showed transgressive segregation, with values ranging from 22.2% to 83.3%. The RGAs also showed transgressive segregation, with values ranging from 35.0° to 79.0°. Broad-sense heritabilities of RDR and RGA in KD-F2 plants were 68.7% and 57.1%, respectively.

**Figure 4:**
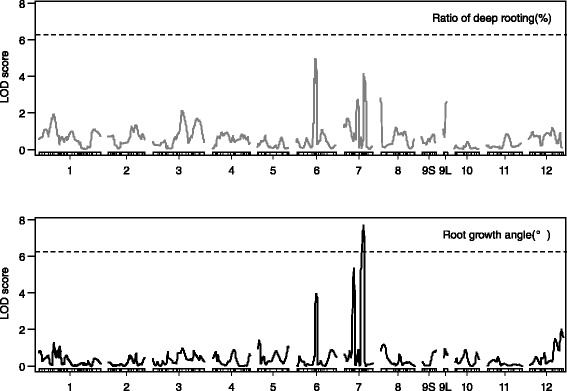
**LOD score curves for the QTLs for the ratio of deep rooting and root growth angle in the KD-F2 plants.** Rectangles represent linkage maps, with the DNA marker positions shown as vertical lines. Chromosome numbers are indicated under each linkage map (short arms are on the left). 9S and 9 L indicate separate linkage maps for the short and long arms of chromosome 9, respectively, for the region around *DRO1* that is homozygous for Kinandang Patong. Dotted lines indicate the LOD thresholds (6.20 for both the ratio of deep rooting and the root growth angle).

Among the 768 SNP markers, 273 (35.5%) were polymorphic between IR64 and Kinandang Patong. To resolve the linkage gaps and to saturate the regions around the LOD support intervals for the QTLs detected with the DNA markers, we added 33 polymorphic SSR markers in these regions. The linkage map for the KD-F2 plants, composed of 306 DNA markers, covered almost the entire rice genome (Additional file 2: Figure S2). The total map length was 1359.8 cM, and the average distance between markers was 4.63 cM. The linkage map of chromosome 9 was divided into two linkage groups near the region of *DRO1* because Dro1-NIL has the same chromosome segment as Kinandang Patong in this region (Figure 4, Additional file 2: Figure S2).

**Figure 5:**
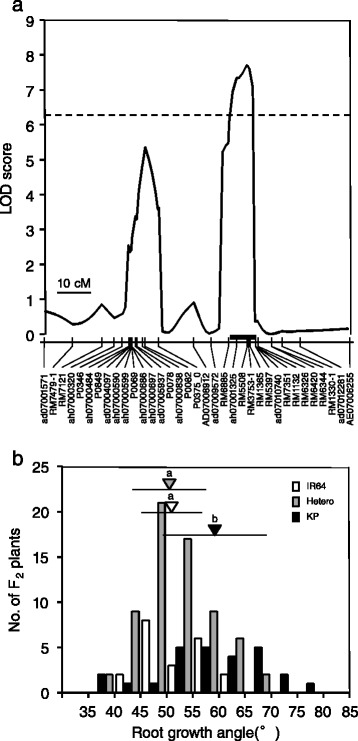
**Chromosomal position and allelic effect of the QTL for root growth angle detected on chromosome 7 in the KD-F2 plants. (a)** The peak of the LOD curve indicates the putative position of a QTL for root growth angle. Vertical lines in the linkage map indicate the genetic positions (cM) of the DNA markers. The black bar above the linkage map indicates 1.8-LOD support intervals calculated by using the lodint function in R/qtl. DNA markers are shown under the linkage maps. The horizontal dotted line indicates the LOD threshold (6.20). **(b)** Frequency distribution for root growth angle in the KD-F2 plants, for three genotypes for the DNA marker (RM5508) closest to the QTL for root growth angle: homozygous for IR64 and Kinandang Patong (KP), or heterozygous. The triangles indicate the position of the mean for each allele, and the horizontal lines indicate the SD. The same shading is used for the triangles and the corresponding bars. The means labeled with different letters differed significantly among the three alleles (*P* < 0.05, Tukey’s multiple-comparison test).

No QTL for RDR was detected at an LOD threshold of 6.2, although two minor QTLs were suggested on chromosomes 6 (LOD = 4.9 at SNP marker ah06000736) and 7 (LOD = 4.1 at SSR marker RM5397) when we decreased the LOD threshold to 3.0 (Figure 4). On the other hand, we detected one QTL for RGA at SSR marker RM5508 on chromosome 7 based on an LOD threshold of 6.2 (Table 1; Figures 4, 5a and Additional file 2: Figure S2). This QTL showed a relatively large contribution to the phenotypic variance, explaining 21.9% of the total (Table 1). The additive effect of the homozygous Kinandang Patong allele at this QTL on RGA was 10.0° (i.e., = 2 × the AE for a single copy of the allele). The mean RGA of lines homozygous for the Kinandang Patong allele at the RM5508 closest to this QTL was significantly larger than that of lines homozygous for the IR64 allele (Figure 5b). When we decreased the LOD threshold to 3.0, we found evidence of two minor QTLs for RGA (Table 1; Figure 4): one on chromosome 6 (LOD = 4.0 at SNP marker ah06000736) and one on chromosome 7 (LOD = 5.4 at SNP marker P0082). A two-dimensional scan produced a heat map that revealed no significant epistatic interaction throughout the genome in this population (Additional file 3: Figure S3).

**Table 1:** **Putative QTLs for root growth angle detected in KD-F2**

**Chr.**	**Nearest marker**	**Map position (Mb)** ^**a**^	**Genetic distance (cM)** ^**b**^	**LOD**	***AE*** **(°)** ^**c**^	***DE*** **(°)** ^**d**^	***R*** ^**2e**^
6	ah06000736	14.01	0.0	4.0	−3.7	1.3	10.2
7	P0082	17.12	0.0	5.4	−4.7	2.7	13.2
7	RM5508	23.56	0.0	7.7*	5.0	−4.2	21.9
							37.2^f^

^a^Physical map position of each marker based on the latest version of the RAP database (IRGSP-1.0; http://rapdb.dna.affrc.go.jp/).

^b^Genetic distance from the QTL LOD peak to the nearest marker.

^c^Additive effect of the allele from Kinandang Patong compared with that from IR64.

^d^Dominance effect of the allele from Kinandang Patong compared with that from IR64.

^e^Percentage of the phenotypic variance explained by each QTL.

^f^Percentage of the phenotypic variance explained by all three QTLs.

*Putative QTL with a significant LOD score based on 1000 permutation tests at the 5% level.

## Discussion

Evaluation of RDRs in the 26 IK-CSSLs showed that only the SL1020 plants had a significantly larger RDR than IR64. SL1020 has the functional allele of *DRO1* derived from Kinandang Patong, whereas all other lines have the non-functional allele derived from IR64. However, the RDR of SL1020 was significantly smaller than that of Kinandang Patong, suggesting that undetected QTLs for RDR may explain the difference of RDR between SL1020 and Kinandang Patong and that they exist in other chromosomal regions. It was unclear why we found no other RDR QTL in the IK-CSSLs. Based on the IK-CSSL results, we hypothesized that there are one or more additional undetected QTLs that can only function in a genetic background that has the functional allele of *DRO1*. These undetected QTLs would affect the genetic pathway of *DRO1*, and therefore should not be identified in IK-CSSLs that have the non-functional allele of *DRO1* (i.e., all IK-CSSLs except SL1020). It was also possible that undetected QTLs were located in some small chromosomal segments that were homozygous for IR64. We therefore created a KD-F2 population derived from a cross between Kinandang Patong and Dro1-NIL, which had the functional allele of *DRO1* in the IR64 genetic background.

We detected no significant QTLs for RDR (i.e., QTLs with a value that exceeded the LOD threshold) in the KD-F2 plants. This result might be explained by our quantification of RDR using the basket method. Dro1-NIL had larger RDR values than IR64. We hypothesize that RDR might be not adequate to evaluate RGA differences among the KD-F2 plants, even though the basket method let us successfully detect a significant difference in RGA between IR64 and Kinandang Patong (Uga et al. 2011). Therefore, we directly measured RGA in the KD-F2 plants and performed QTL analysis based on this data. This approach revealed a significant QTL for RGA on chromosome 7 (Table 1). The additive effect of the homozygous allele for Kinandang Patong at this QTL (RGA = 10°) can explain the difference of RGA between Dro1-NIL and Kinandang Patong (RGA = 8.8°). This suggests that we can reproduce the deep roots of Kinandang Patong in the IR64 genetic background by introducing this QTL from Kinandang Patong into Dro1-NIL. We then hypothesized that one or more key genes responsible for RGA may be located in this chromosome region. We identified *DRO1* and *DRO2* in our previous studies (Uga et al. 2011, 2013b). Thus, we propose designating this third QTL for deeper rooting as *DEEPER ROOTING 3* (*DRO3*).

Detection of *DRO3* in the KD-F2 plants but not in the IK-CSSLs supports the hypothesis that *DRO3* affects the genetic pathway of *DRO1*. To test this hypothesis, we are currently developing two types of NILs for *DRO3*: one in IR64 (which has the non-functional allele of *DRO1*) and one in Dro1-NIL (which has the functional allele of *DRO1*). We hypothesize that *DRO3* was not detected in our previous research in the IK-RILs that contain *DRO1* because segregation of major QTLs often conceals the genetic effects of minor QTLs in primary mapping populations such as F_2_ and RIL populations (Fukuoka et al. 2010). We expected to detect *DRO2* in the KD-F2 plants because *DRO2* has a large genetic effect and was found in three other F_2_ populations derived from shallow-rooting varieties that had the functional allele of *DRO1*, with Kinandang Patong (Uga et al. 2013b). We hypothesized that the same allele or a small difference in the genetic effects at *DRO2* between IR64 and Kinandang Patong was not involved in the natural variation of RGA between IR64 and Kinandang Patong. However, the present QTL results and our previous reports (Uga et al. 2013a,b) suggest that *DRO1*, *DRO2*, and *DRO3* are associated with deep rooting of Kinandang Patong.

*DRO3* was detected near *qSOR1*, but comparison of the physical map positions between the 1.8-LOD support intervals for *DRO3* (23.00 to 23.95 Mb) and the candidate region for *qSOR1* (24.78 to 25.59 Mb) based on the Nipponbare genome sequence in the RAP database (http://rapdb.dna.affrc.go.jp/) showed that they had different locations on chromosome 7. Since there is no reported QTL for RGA in the *DRO3* region, we compared the positions of *DRO3* and the other root QTLs summarized by Courtois et al. (2009). According to their report, many QTLs for the weight of deep roots, maximum root length, root thickness, and root dry weight were located in the *DRO3* region. Further study using a *DRO3* NIL will be needed to elucidate whether *DRO3* shows pleiotropic effects on other root morphological traits. We identified no genes homologous to *DRO1* in the *DRO3* region. Next, we surveyed known root genes characterized in mutants in the *DRO3* region by using the Overview of functionally characterized Genes in Rice Online database (OGRO, http://qtaro.abr.affrc.go.jp/ogro; Yamamoto et al. 2012). We also checked the *DRO3* region for the presence of auxin-related genes, because many auxin-related genes such as *CROWN ROOTLESS 1*(*CRL1*)/*ADVENTITIOUS ROOTLESS 1* (Inukai et al. 2005; Liu et al. 2005), *OsPID1* (Morita and Kyozuka 2007), *CRL4*/*OsGNOM1* (Kitomi et al. 2008; Liu et al. 2009), *CRL5* (Kitomi et al. 2011), and *OsIAA13* (Kitomi et al. 2012) are involved in root gravitropism, which determines RGA. However, we found no candidate root- or auxin-related genes in the *DRO3* region, indicating that *DRO3* appears to be a novel gene that controls RGA in rice. Therefore, we are currently carrying on map-based cloning of *DRO3* in our laboratory to clarify the genetic mechanisms of *DRO3* and the relationship of the molecular functions between *DRO1* and *DRO3*.

We found several CSSLs with agronomic traits that differed significantly from those of IR64 in the paddy field (Additional file 1: Figure S1), although only one of the IK-CSSL lines (SL1020) had a significantly larger RDR than IR64. These results suggest that the IK-CSSLs are useful genetic material for discovering new QTLs for other traits. In our previous research, we found wide genetic variation in aspects of root system architecture other than RGA, such as root length, thickness, and volume, between IR64 and Kinandang Patong under upland field conditions (Uga et al. 2008, 2013a). We expect to detect new QTLs for other root morphological traits in the IK-CSSLs. For this purpose, we are currently trying to evaluate other root traits using the IK-CSSLs. Identification of undetected QTLs for root traits in the IK-CSSLs will accelerate marker-assisted selection for characteristics of the root system architecture, which will in turn support breeding for improved drought avoidance in rice.

## Conclusions

We discovered new QTL for RGA on chromosome 7 (*DRO3*) explaining the difference in RGA between IR64 and Kinandang Patong other than *DRO1*. Our genetic analyses suggest that *DRO3* may be a promising QTL that can be used to improve RGA in IR64. We believe that development of a gene pyramiding line that contains the Kinandang Patong alleles of both *DRO1* and *DRO3* in the IR64 genetic background will produce plants that avoid drought stress better than Dro1-NIL. Cloning of *DRO3* may also accelerate molecular breeding for higher RGA in other shallow-rooting varieties.

## Methods

### Plant materials

To detect new QTLs associated with genetic variation in RGA between IR64 and Kinandang Patong, we developed a set of 26 chromosome segment substitution lines derived from a cross between IR64 and Kinandang Patong. (Additional file 4: Figure S4) summarizes the procedure for development of the IK-CSSLs. Kinandang Patong was crossed with IR64 to produce F_1_ plants. The F_1_ plants were then backcrossed four times with IR64 to obtain BC_4_F_1_ plants. In each backcrossed generation, plants heterozygous for the target regions were selected by using DNA markers to guide further backcrossing or self-pollination. BC_4_F_1_ plants were consecutively self-pollinated three times to substitute target regions homozygous for the donor cultivar Kinandang Patong allele and those homozygous for the recurrent parent cultivar IR64 at other regions.

Based on our analysis of the results obtained with the IK-CSSLs, we also developed an F_2_ population (KD-F2) consisting of 121 lines derived from a cross between Kinandang Patong and Dro1-NIL, which is a NIL homozygous for the Kinandang Patong allele of *DRO1* in the genetic background of IR64 (Uga et al. 2013a).

We surveyed the genotypes of the IK-CSSLs and KD-F2 by using the markers described in the section “DNA marker analysis”.

### Measurements of shoot physiological and morphological traits in the IK-CSSLs

Heading date, culm length, and the length and number of panicles in the IK-CSSLs and IR64 were evaluated in paddy field located at the National Institute of Agrobiological Sciences (36°1’N, 140°6’E) in Tsukuba, Japan, in the summer of 2012. Seeds were sown in the seedling nursery on April 16 and 60 plants in each CSSL per plot were transplanted on May 16. Three plots per line were arranged in a randomized block design. We determined the number of days from sowing to heading of the first panicle as heading date. After maturating, we measured the culm length, and the length and number of panicles in each plant. Twenty-seven samples (9 plants × 3 plots) were used to calculate means in each line.

### Measurements of the ratio of deep rooting (RDR) and RGA

RDR was evaluated quantitatively in the IK-CSSLs and KD-F2 based on a basket method that uses open stainless-steel-mesh baskets filled with soil in a greenhouse (average air temperature, 30°C; average relative humidity, 50%; natural lighting), as described previously (Uga et al. 2011). We defined the RDR as the number of roots that penetrated the lower part of the mesh (i.e., ≥50° from the horizontal, centered on the stem) divided by the total number of roots that penetrated the whole mesh. Ten samples were used to calculate mean RDR in each line of IK-CSSLs. After measurement of RDR in the KD-F2 plants, the roots in each basket were washed carefully, and the RGA of each plant was determined by measuring the angle between the soil surface (horizontal line) and the shallowest primary roots with a protractor, as described previously (Uga et al. 2013a).

### DNA marker analysis

We determined the genotypes of the IK-CSSLs by using 394 single-nucleotide polymorphism (SNP) markers selected from genome-wide SNP marker data (Yonemaru et al. 2014), and 125 simple sequence repeat (SSR) markers selected on the basis of the data from the International Rice Genome Sequencing Project (2005). We also determined the genotypes of the KD-F2 by using 273 SNP and 33 SSR markers. Total DNA was extracted from leaves by using the CTAB method (Murray and Thompson 1980). The polymorphic SNP markers were detected from a set of 768 custom SNP panels by using a GoldenGate Genotyping Universal-32 768-plex Assay Kit and the BeadStation 500G system (both from Illumina, San Diego, CA, USA) according to the manufacturer’s instructions. PCR amplification for SSR analysis was performed in 5-μL reaction mixtures containing 0.5 μL (20 ng) DNA, 1.0 μL 5× PCR buffer, 0.1 μL 10 mM dNTPs, 0.025 μL (5 units) of KAPA2G Fast DNA Polymerase (Kapa Biosystems, Boston, MA, USA), 0.125 μL of a mixture of forward and reverse primers (20 pM each), and 3.25 μL H_2_O. The PCR program consisted of an initial denaturation for 1 min at 95°C; followed by 35 cycles of 10 s at 95°C, 10 s at 55°C, and 1 s at 72°C; and with a final extension for 30 s at 72°C. PCR products were separated by electrophoresis in 3% agarose gels (Agarose LE; Promega Corporation, Madison, WI, USA) at 200 V for 80 min.

### Statistical and QTL analyses

To compare the mean RDRs of the 26 IK-CSSLs and IR64, we used Dunnett’s test in version 7.0 of the JMP software (SAS Institute, Cary, NC, USA). All lines were compared with IR64 as the reference.

The broad-sense heritability (*h*^2^_B_) of RGA in KD-F2 population, *h*^2^_B_ [F_2_], was estimated from the variances of the parental plants and the variance among the F_2_ lines, and was calculated as follows:

$$ {h^2}_B\left[{F}_2\right]=\left({V}_p\left[{F}_2\right]\hbox{--} \left\{{V}_p\left[{P}_1\right]+{V}_p\left[{P}_2\right]\right\}/2\right)/{V}_p\left[{F}_2\right] $$

where *V*_p_[F_2_] is the phenotypic variance among the F_2_ lines, and *V*_p_[P_1_] and *V*_p_[P_2_] are the phenotypic variances of parental plants 1 and 2.

Construction of a linkage map and QTL analysis for the KD-F2 plants were performed using the R/qtl software (http://www.rqtl.org/; Broman et al. 2003). Genetic distances were estimated using the software’s Kosambi map function (Kosambi 1944). Putative QTLs were detected using the composite interval mapping (CIM) function. The CIM threshold was based on the results of 1000 permutations at a 5% significance level (Churchill and Doerge 1994). The confidence intervals around each significant QTL peak were determined using 1.8-LOD support intervals (Broman et al. 2003). The additive and dominant effects and the percentage of phenotypic variance explained by each QTL (*R*^*2*^) at the maximum LOD score were estimated using the sim.geno, makeqtl, and fitqtl functions in R/qtl (Broman et al. 2003). To identify interactions between QTLs, two-dimensional scans with a two-QTL model were conducted in R/qtl with the thresholds based on the results of 1000 permutations at a 5% significance level (Broman et al. 2003).

To compare the mean RGAs in three genotypes for the DNA marker (RM5508) closest to the RGA QTL in the KD-F2, the Tukey’s multiple-comparison test provided by JMP version 7.0 was used.

## Additional files

Additional file 1: **Shoot physiological and morphological traits of the 26 IK-CSSLs and of IR64.**

Additional file 2: **Position of the markers nearest to the QTL for root growth angle detected in the KD-F2 plants.**

Additional file 3: **Heat map for the two-dimensional genome scan with a two-QTL model in the KD-F2 plants.**

Additional file 4: **Illustration of the scheme used to develop chromosome segment substitution lines (IK-CSSLs) carrying Kinandang Patong chromosome segments in the IR64 genetic background.**
