# Supplementary material for: A QTL for root growth angle on rice chromosome 7 is involved in the genetic pathway of DEEPER ROOTING 1
Source: Rice (N Y). 2015 Feb 5;8:8. doi: 10.1186/s12284-015-0044-7 (PMC4384719; doi:10.1186/s12284-015-0044-7)
Supplement: Supplementary file 1 — Shoot physiological and morphological traits of the 26 IK-CSSLs and of IR64. [file 12284_2015_44_MOESM1_ESM.pdf]

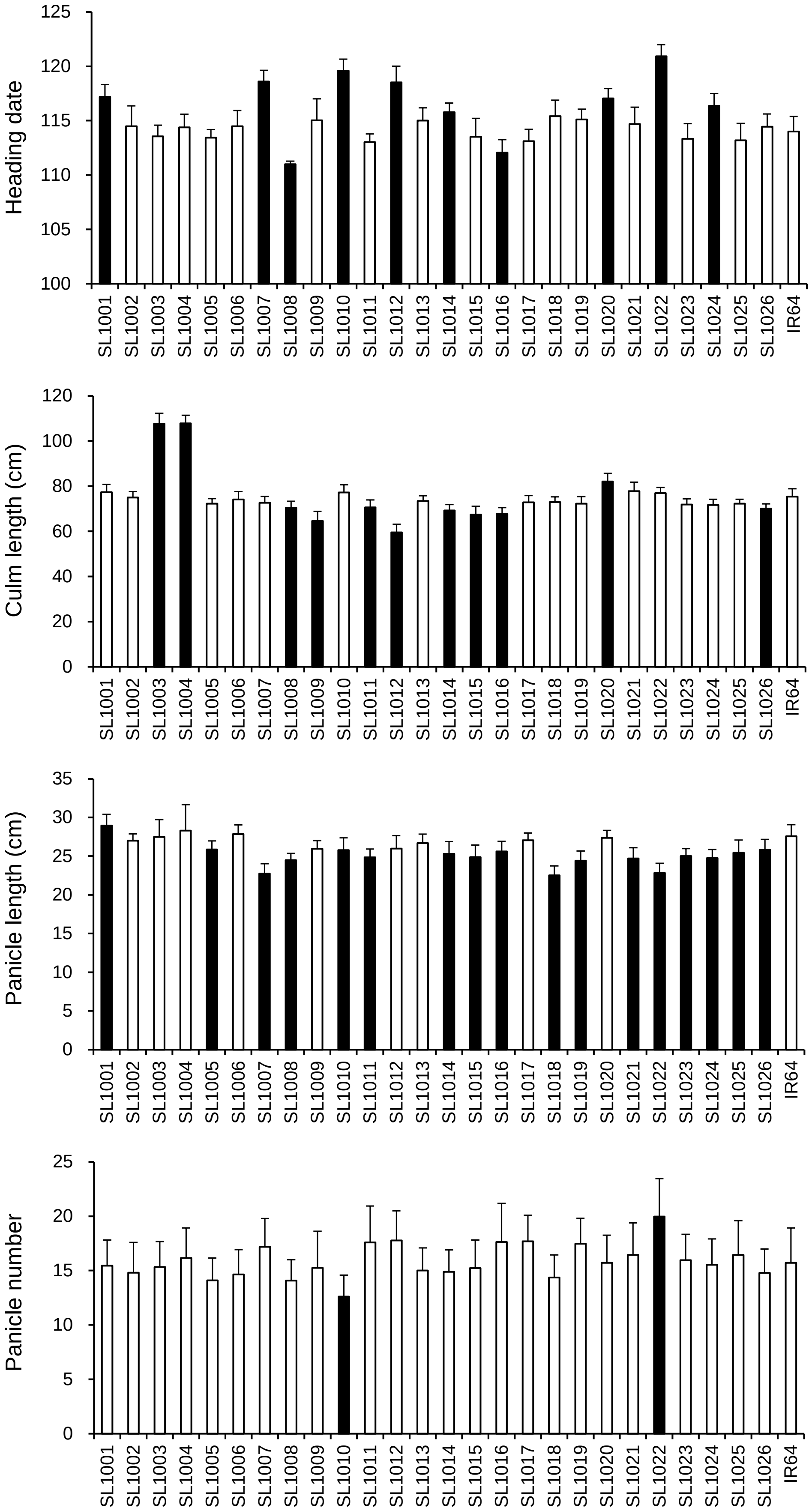

Figure S1. Shoot physiological and morphological traits of the 26 IK-CSSLs and of IR64.

Values are means+SD (n = 27) for each line. Black bars indicate a significant difference between the IK-CSSL and IR64 ($p < 0.001$, Dunnett's test).
