# Supplementary material for: A QTL for root growth angle on rice chromosome 7 is involved in the genetic pathway of DEEPER ROOTING 1
Source: Rice (N Y). 2015 Feb 5;8:8. doi: 10.1186/s12284-015-0044-7 (PMC4384719; doi:10.1186/s12284-015-0044-7)
Supplement: Supplementary file 2 — Position of the markers nearest to the QTL for root growth angle detected in the KD-F2 plants. [file 12284_2015_44_MOESM2_ESM.pdf]

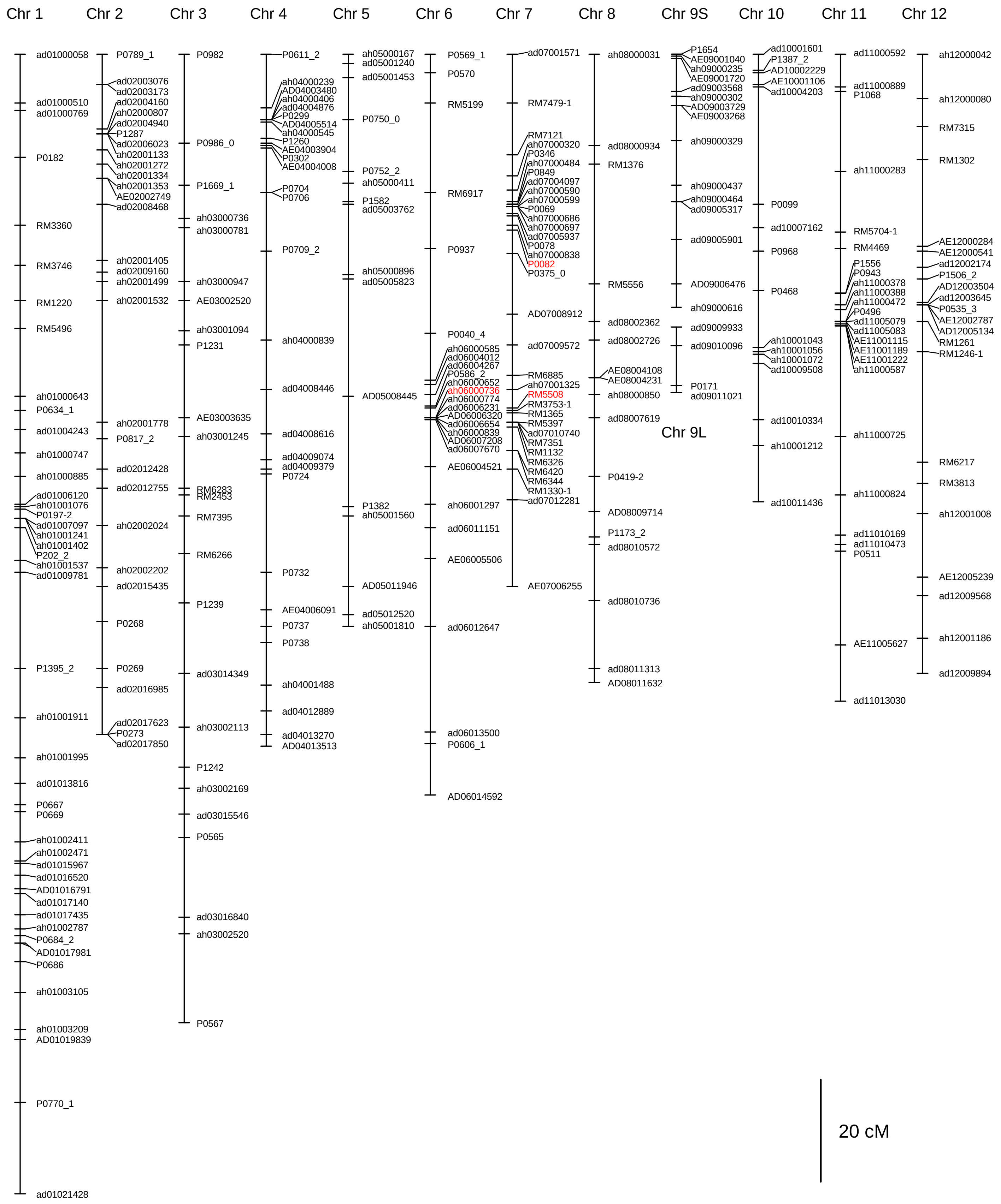

Figure S2. Position of the markers nearest to the QTL for root growth angle detected in the KD-F2 plants.
Chromosome numbers are indicated above each linkage map. Marker names are indicated to the right of each linkage map. The red markers are the ones nearest to the putative QTLs described in Table 1. For chromosome 9, 9S represents the short arm and 9L represents the long arm.
