# Supplementary material for: A QTL for root growth angle on rice chromosome 7 is involved in the genetic pathway of DEEPER ROOTING 1
Source: Rice (N Y). 2015 Feb 5;8:8. doi: 10.1186/s12284-015-0044-7 (PMC4384719; doi:10.1186/s12284-015-0044-7)
Supplement: Supplementary file 3 — Heat map for the two-dimensional genome scan with a two-QTL model in the KD-F2 plants. [file 12284_2015_44_MOESM3_ESM.pdf]

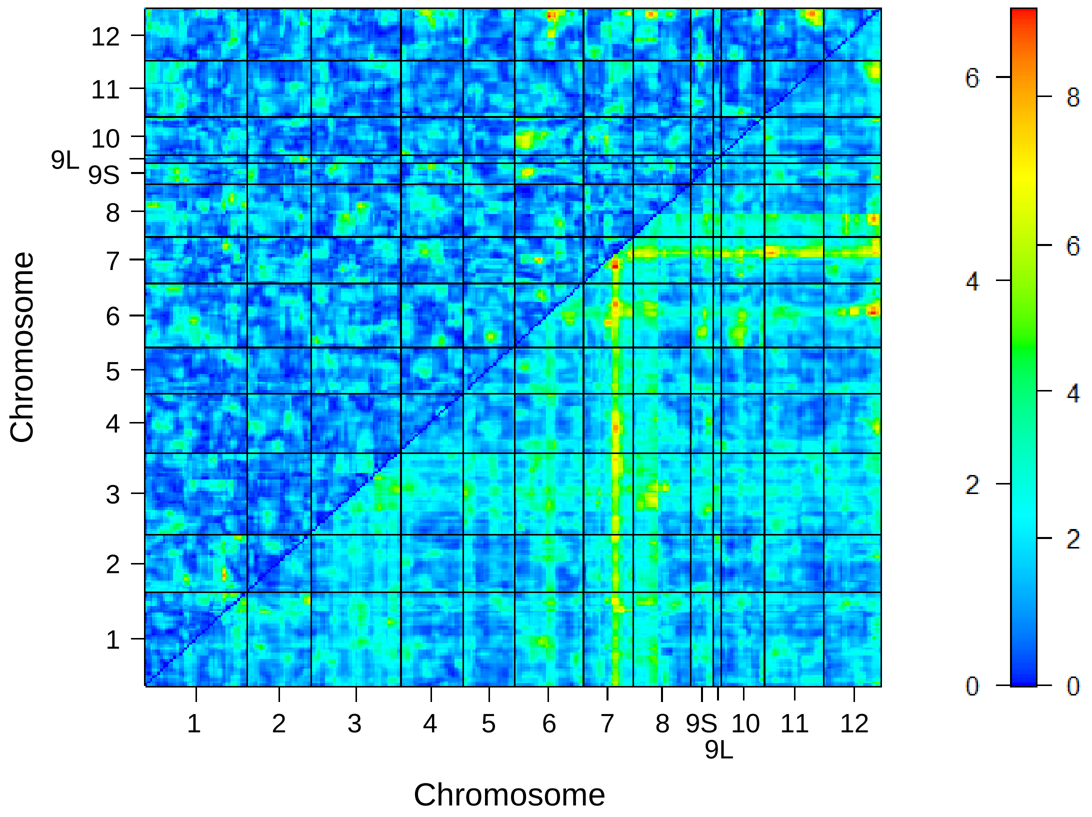

Figure S3. Heat map for the two-dimensional genome scan with a two-QTL model in the KD-F2 plants.

Top left triangle: the maximum LOD scores for the interaction model. Bottom right triangle: the maximum LOD scores for the full model (two QTLs plus an interaction). The color scale indicates the values for the interaction model on the left (LOD threshold = 6.52) and the full model on the right (LOD threshold = 9.03).
