# Supplementary material for: A QTL for root growth angle on rice chromosome 7 is involved in the genetic pathway of DEEPER ROOTING 1
Source: Rice (N Y). 2015 Feb 5;8:8. doi: 10.1186/s12284-015-0044-7 (PMC4384719; doi:10.1186/s12284-015-0044-7)
Supplement: Supplementary file 4 — Illustration of the scheme used to develop chromosome segment substitution lines (IK-CSSLs) carrying Kinandang Patong chromosome segments in the IR64 genetic background. [file 12284_2015_44_MOESM4_ESM.pdf]

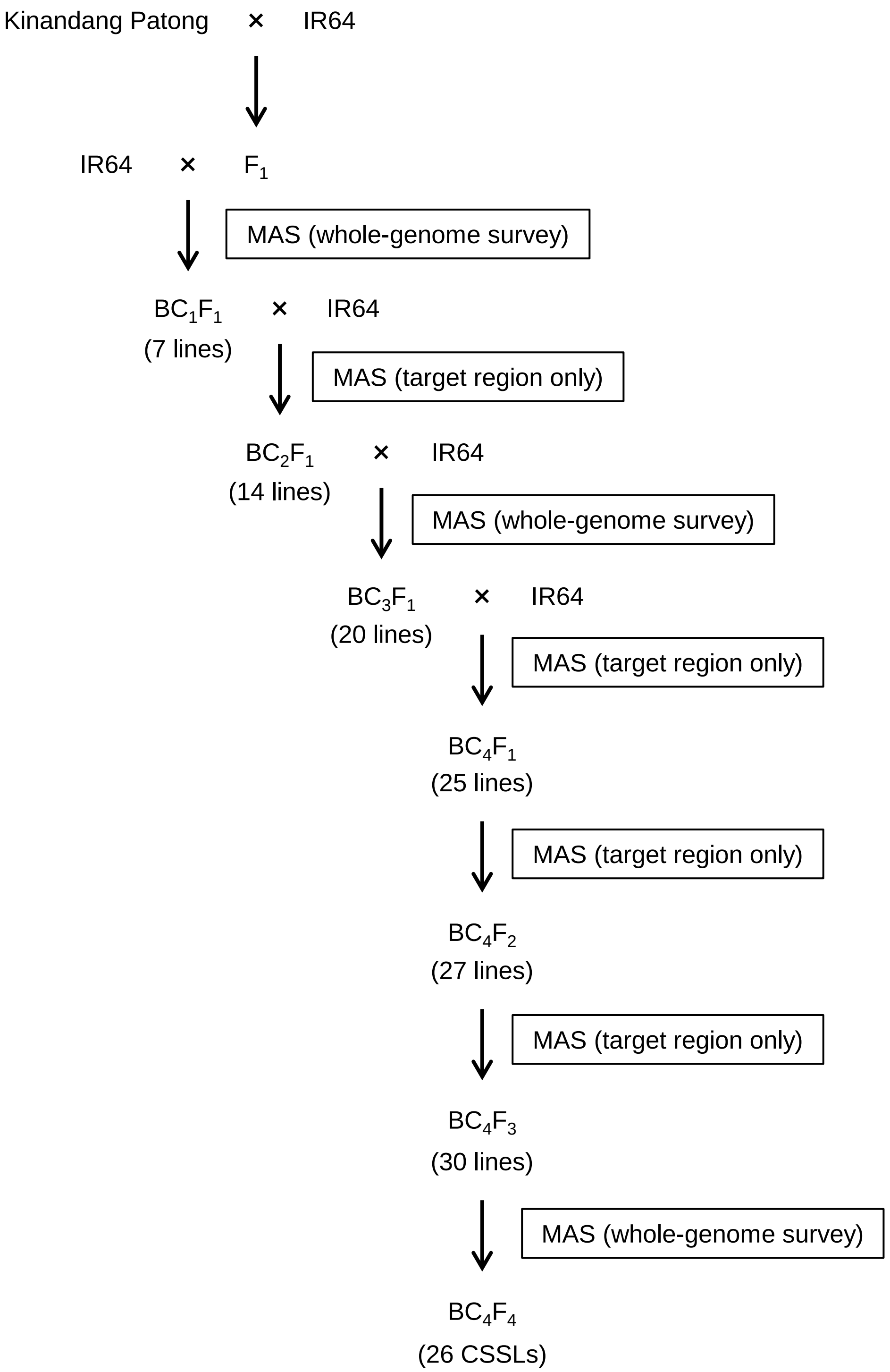

Figure S4. Illustration of the scheme used to develop chromosome segment substitution lines (IK-CSSLs) carrying Kinandang Patong chromosome segments in the IR64 genetic background. MAS, marker-assisted selection.
